# Genetic diversity of *Hepatozoon* spp. in rodents from Brazil

**DOI:** 10.1038/s41598-019-46662-2

**Published:** 2019-07-12

**Authors:** L. Perles, A. L. R. Roque, P. S. D’Andrea, E. R. S. Lemos, A. F. Santos, A. C. Morales, R. Z. Machado, M. R. André

**Affiliations:** 1 Laboratório de Imunoparasitologia, Departamento de Patologia Veterinária, Faculdade de Ciências Agrárias e Veterinárias, Universidade Estadual Paulista “Júlio de Mesquita Filho” (FCAV/UNESP), Jaboticabal, SP, Brazil; 2 Laboratório de Biologia de Tripanosomatídeos, Instituto Oswaldo Cruz/Fiocruz, Rio de Janeiro, RJ, Brazil; 3 Laboratório de Biologia e Parasitologia de Mamíferos Silvestres Reservatórios, Instituto Oswaldo Cruz/Fiocruz, Rio de Janeiro, RJ, Brazil; 4 Laboratório de Hantaviroses e Rickettsioses, Instituto Oswaldo Cruz/Fiocruz, Rio de Janeiro, RJ, Brazil; 5 Departamento de Biologia Aplicada à Agropecuária, Faculdade de Ciências Agrárias e Veterinárias, Universidade Estadual Paulista “Júlio de Mesquita Filho” (FCAV/UNESP), Jaboticabal, SP, Brazil

**Keywords:** Parasite evolution, Parasite genetics, Parasitic infection, Pathogens

## Abstract

*Hepatozoon* spp. are Apicomplexan protozoa that parasitize a wide diversity of vertebrate hosts. In Brazil, few studies have reported the occurrence of *Hepatozoon* spp. in rodent species. Additionally, an evaluation of the population structure and distribution of *Hepatozoon* species over several Brazilian biomes has not yet been performed. The present work aimed to investigate the genetic diversity of *Hepatozoon* spp. in rodents from 31 genera sampled in five Brazilian biomes. Samples were submitted to PCR assays for *Hepatozoon* spp. targeting two regions of the 18S rRNA gene. Infection by *Hepatozoon* spp. was detected in 195 (42.2%) rodents comprising 24 genera. Phylogenetic analyses of 18S rRNA sequences grouped all sequences in the clade of *Hepatozoon* spp. previously detected in rodents and reptiles, apart from those detected in domestic/wild carnivores. These data raise two non-exclusive hypotheses: (i) rodents play an important role as intermediate or paratenic hosts for *Hepatozoon* infections in reptiles; and (ii) rodents do not seem to participate in the epidemiology of *Hepatozoon* infections of domestic/wild canids and felids in Brazil. TCS analyses performed with available 18S rRNA *Hepatozoon* sequences detected in rodents from Brazil showed the occurrence of six haplotypes, which were distributed in two large groups: one from rodents inhabiting the coastal region of Brazil and Mato Grosso state, and another from rodents from the central region of the country. A wide survey of the South American territory will help to elucidate the evolutionary history of *Hepatozoon* spp. parasitizing Rodentia in the American continent.

## Introduction

The genus *Hepatozoon* belongs to one of the six genera of blood parasites known as hemogregarines, which have a heteroxenous life cycle involving an intermediate vertebrate host and a blood feeding definitive invertebrate host. Studies have detected the presence of *Hepatozoon* species in domestic and wild mammals, birds, reptiles, and amphibians^1^.

The epidemiology of hepatozoonosis in wild and domestic animals in Brazil has not been fully elucidated. The role of rodents in the epidemiology of *Hepatozoon* spp. warrants investigation to evaluate whether genotypes of *Hepatozoon* spp. circulating in rodents are specific to this group of mammals, or if there are genotypes shared between this group of mammals and wild carnivores or reptiles. In the latter scenario, rodents might play an important role as intermediate or paratenic hosts for species of *Hepatozoon*.

Predation might represent an important transmission route for Hepatozoidae protozoa. For instance, predation is an important route for reptile infections by *Hepatozoon ayorgbor*. Experimental transmission to snakes was achieved when these animals were fed rodent tissues infected with *H*. *ayorgbor*^2^. For mammals, dogs from rural areas often live in areas surrounding woods and might predate *Hepatozoon*-infected rodents^3^. Several studies performed in the USA have demonstrated that *H*. *americanum* can be transmitted by predation of rodents and lagomorphs^4–6^. In the African continent, Maia^7^ suggested that wild carnivores can become infected by *Hepatozoon* sp. through predation of rodents.

Despite the molecular confirmation of *Hepatozoon* infecting rodents in several regions around the world, studies aiming at analyzing the genetic diversity and phylogeography of these apicomplexan protozoa are scarce. In Brazil, few studies have reported the occurrence of *Hepatozoon* spp. in rodent species^8–12^. Additionally, the population structure, the distribution of *Hepatozoon* species over several Brazilian biomes, and how it reflects into the parasite evolution have not yet been assessed. The present study aimed to investigate the occurrence of *Hepatozoon* spp. in wild rodents from several regions of Brazil. Additionally, this work aimed to evaluate the genetic diversity of haplotypes in sampled rodents to draw genetic and geographical inferences across the Brazilian territory.

## Results

Among the 472 rodent spleen samples analyzed, 462 were positive for the amplification of a fragment of the *irbp* gene (endogenous control of reaction). The ten samples negative for the *irbp*-PCR assay were also negative in the cPCR based on the *gapdh* gene and were excluded from subsequent analysis. The average concentration of DNA was 154.62 ng/µL (0.1 to 812.0 ng/µL), and the 260/280 ratio was 1.95 (0.7 to 6.18).

Of the 462 rodents analyzed, 195 (42.2%) were considered positive for *Hepatozoon* spp., amplifying in one or both regions of 18SrRNA gene (based on protocols described by Perkins and Keller^13^ and Ujvari^14^) (Supplementary Information, Table 1). Amplicons from nine positive samples (9/195- 4,61%) for both protocols were sequenced and used for concatenated analyzes.

Of the 462 rodents analyzed, 69 (14.93%) were positive for *Hepatozoon* spp. based on the first 18SrRNA gene region analyzed (according to the protocol described by Perkins and Keller^13^) (Supplementary Information, Table 1). Amplicons from seventeen positive samples (17/69- 24.6%) were sequenced. The sequences of 18SrRNA *Hepatozoon* spp. obtained from seven rodents showed 99–100% identity with *Hepatozoon* sp. detected in the reptile *Tarentola deserti* in Africa (KU680460) by BLAST analysis. Eight sequences showed 100% identity with *Hepatozoon* sp. detected in *Amblyomma fuscum* (KU955319) collected from a rodent *Akodon montensis* in Santa Catarina, south Brasil. One sample showed 99% identity with *Hepatozoon* sp. detected in a rodent *Oecomys marmorae* (KX776332) sampled in Brazilian Pantanal. Lastly, one sample showed 99% identity with *Hepatozoon* sp. from a *Caiman crocodilus* (MF322539) sampled in Brazilian Pantanal.

Of the 462 rodents analyzed, 172 (37.2%) were positive for *Hepatozoon* spp. based on the second 18SrRNA gene region analyzed (according to the protocol based on the 18S rRNA gene described by Ujvari^14^) (Supplementary Information, Table 1). Nineteen of these positive samples (19/172, 11.04%) were sequenced. The sequences of 18SrRNA *Hepatozoon* spp. obtained from 16 rodents (MH111405, MH111406, MH111407, MH111408, MH111409, MH111410, MH111411, MH111412, MH111413, MH111415, MH111416, MH111417, MH111419, MH111420, MH111422, MH111423) showed 98–100% identity with *Hepatozoon* sp. detected in the rodent *Akodon sp*. (KU667308) sampled in Brazil (Botucatu, São Paulo state). One sequence (MH111418) showed 100% identity with *Hepatozoon* sp. detected in a snake (*Psammophis schokari*) (KX453646) sampled in Omã, on the southeastern coast of the Arabian Peninsula. One sequence (MH111404) showed 100% identity with *Hepatozoon* sp. detected in a snake (*Phymaturus calcogaster*) (KX387861) sampled in Spain. Lastly, one sample (MH111421) showed 100% identity with *Hepatozoon* sp. detected in a rodent (*Thylamys macrurus*) (KX776354) sampled in Pantanal, Mato Grosso do Sul, Brazil.

**Figure 1:**
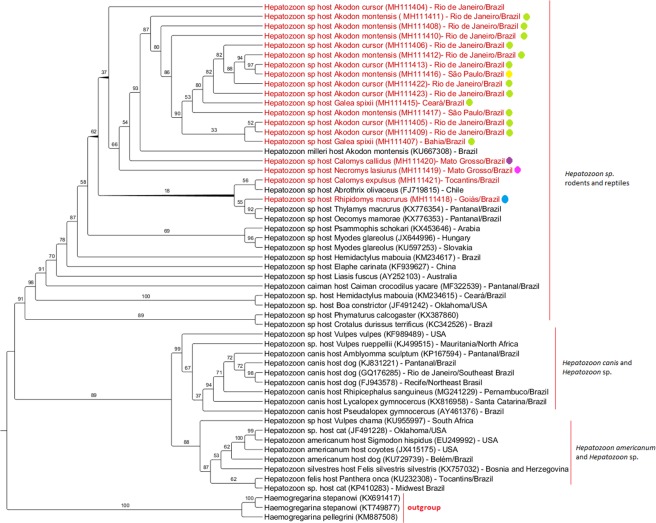
Phylogenetic tree based on an alignment of 600 bp fragment (Uvjari *et al*., 2004) of *Hepatozoon* spp. 18SrRNA sequences, using ML method and TIM1 + I + G evolutionary model. Numbers at nodes correspond to bootstrap. Accession numbers are indicated in the sequences. Sequences of *Hepatozoon* spp. detected in the present study are highlighted in red. Colored circles indicated in the sequences represent the haplotypes detected.

The phylogenetic inferences estimated by both Maximum Likelihood (ML) and Bayesian methods of *Hepatozoon* spp. 18SrRNA sequences obtained from both PCR protocols separately and concatenated presented similar results (concatenated phylogenetic inference is shown in Supplementary Information, Fig. 1). The sequences clustered in three large branches: (i). The first group comprised *Hepatozoon* sequences detected in rodents and reptiles; (ii). The second grouped sequences of *H*. *canis* and *Hepatozoon* spp. detected in domestic and wild canids; (iii). The third branch composed of *H*. *americanum* and *Hepatozoon* spp. detected in domestic and wild felids and canids. The clades showed considerable statistical support. *Haemogregarina* spp. sequences were used as an outgroup. Figure 1 shows a phylogenetic tree based on an alignment of 600 bp fragment of *Hepatozoon* spp. 18SrRNA using sequences detected in this study and other sequences deposited in GenBank (using ML method).

**Figure 2:**
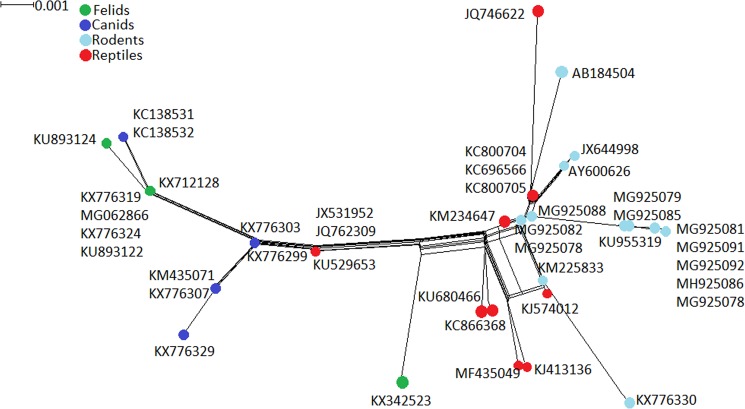
Network analysis of *Hepatozoon* 18S rRNA sequences (Perkins and Keller protocol^13^) obtained from rodents sampled in the present study, compared to previously detected protozoan sequences in reptiles, felids and canids (sequences deposited in GenBank). The analysis was performed with Splitstree software using the parameters “Neighbor-Net and” Uncorrected p-distance”.

Additionally, Splitstree results of *Hepatozoon* spp. 18SrRNA sequences obtained from both PCR protocols showed a high degree of heterogeneity revealing two major clusters: (i) *Hepatozoon* spp. sequences obtained from rodents and reptiles; (ii) *Hepatozoon* spp. sequences obtained from canids and felids (Fig. 2).

**Figure 3:**
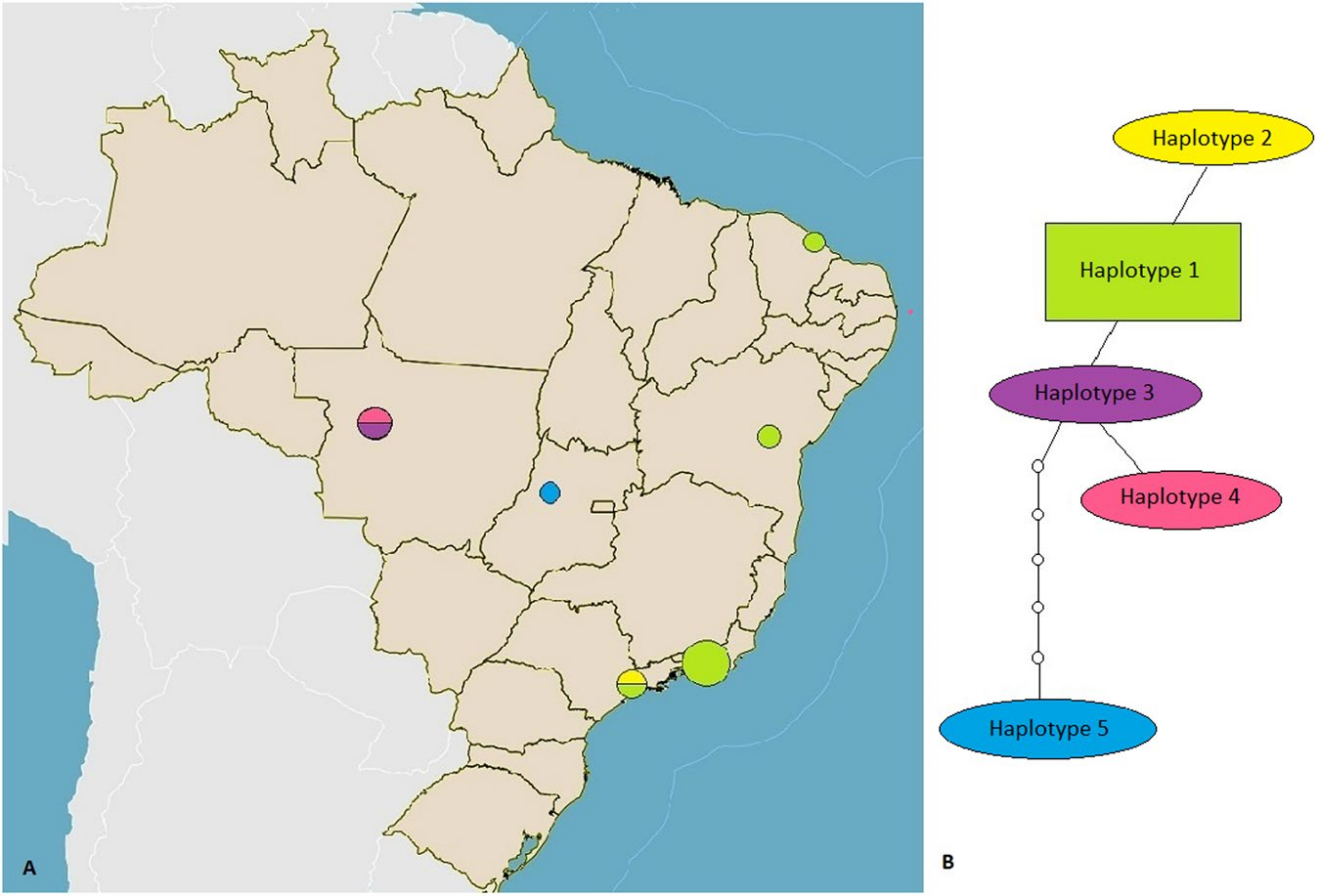
(**A**) Geographical distribution. (**B**) TCS haplotype network of 18S rRNA *Hepatozoon* detected in the present study in rodents from Brazilian biomes.

Nucleotide polymorphisms and DNA divergence between the sequences obtained from this study were analyzed. For this purpose, the obtained sequences were initially aligned with MAFFT software (version 7)^15^. Only sequences with perfect alignment were used for these analyses. The *Hepatozoon* sequences obtained from the two PCR protocols were analyzed separately because they amplify different regions of the 18SrRNA gene. For the first fragment^13^, thirteen sequences presented good alignment (fragment of 185 bp). This fragment showed two haplotypes, with haplotype diversity (Hd) = 0.1538 and number of variable sites (S) = 2. For the second fragment^14^, seventeen sequences presented good alignment (fragment of 277 bp). This fragment showed a higher diversity, with five haplotypes [haplotype diversity (Hd) = 0.426], nucleotide diversity (Pi) = 0,00453 and number of variable sites (S) = 9. Haplotype #1 was the most geographically distributed and was detected in a relatively higher number of rodent species present in the states of São Paulo, Rio de Janeiro, Bahia, and Ceará. Haplotype #2 was represented by one sequence detected in the state of São Paulo. Similarly, haplotypes #3, #4, and #5 were represented by one sequence each, detected in the states of Mato Grosso and Goiás state (#5) (Fig. 3).

**Figure 4:**
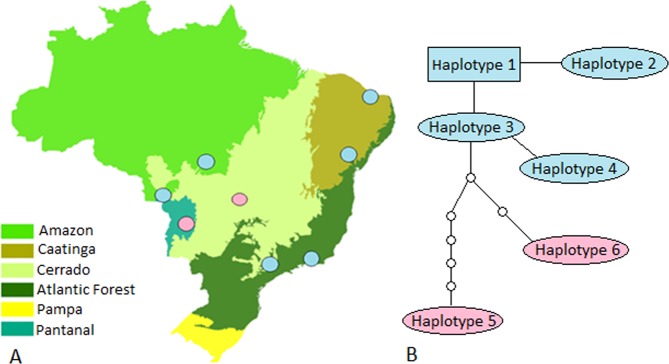
(**A**) Geographical distribution of *Hepatozoon* spp. 18S rRNA haplogroups in Brazilian territory. The colors of the circles correspond to the colors of the haplogroups observed in the network. (**B**) Haplotypic network of *Hepatozoon* sp. generated through mitochondrial gene 18S rRNA sequencing with TCS v.1.21 software^30^. Each line in the network represents a single mutational step; Small circles indicate hypothetical haplotypes that are necessary intermediates among the identified haplotypes, but which were not observed in the sampling. Haplogroup 1 is represented in blue and haplogroup 2 is represented in pink (Fig. 5).

**Figure 5:**
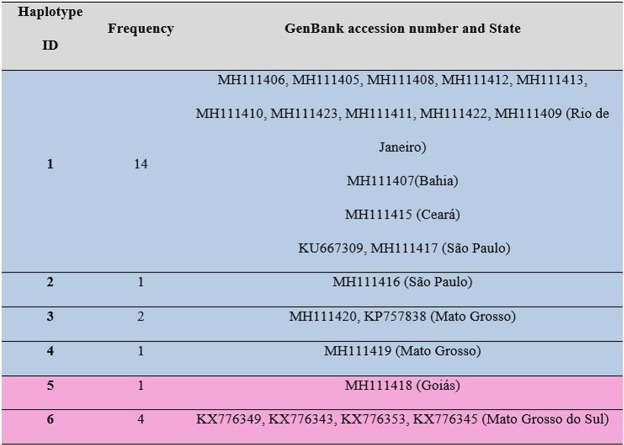
Haplotype identification, frequency and GenBank accession number related to *Hepatozoon* 18S rRNA sequences detected in rodents from different geographical regions in Brazil (Fig. 4). Haplogroup 1 is represented in blue and haplogroup 2 is represented in pink.

Additionally, a haplotype network analysis was carried out using *Hepatozoon* spp. 18S rRNA sequences detected in rodents from other studies performed in Brazil^8–10^. Twenty-six sequences were selected, and the analysis was performed with the TCS software v.1.21^16^ (Fig. 4). These sequences were select due to the perfect alignment presented, resulting in a fragment of 275 bp. Through the TCS analysis, six haplotypes were detected, and these were grouped into two large haplogroups: one from the coastal region of Brazil and sequences from Mato Grosso and another from the central region of Brazil (Goiás and Mato Grosso do Sul). Haplotype #1 was represented by 14 sequences (detected in rodents in São Paulo, Rio de Janeiro, Bahia and Ceará States), haplotype #2 with one sequence (São Paulo), haplotype #3 with two sequences from Mato Grosso, #4 with one sequence from Mato Grosso, #5 with one sequence (Goiás), haplotype #6 with sequences from Mato Grosso do Sul (Figs 4 and 5). Three sequences from Mato Grosso do Sul detected in *Thrichomys fosteri* (KX776351, KX776337, KX776344) were not linked with the other sequences and were grouped separately, forming another haplogroup (using a 95% parsimony threshold).

An analysis of molecular variance (AMOVA) was performed to evaluate the degree of genetic structure among *Hepatozoon* sp. haplogroups. We tested one hierarchy construction segregating the haplogroups according to geographic location, one from the coastal region of Brazil and Mato Grosso state and another from the central region of Brazil (Fig. 4). The results of this analysis were significant (p < 0.05) and the FST value (0.7267) indicated a high degree of genetic structure among the haplogroups (Table 1).

**Table 1:** AMOVA test results. Each haplogroup (haplogroup 1 and 2) was considered as a population.

Type of variation	Variance component	% Variation	FST
Among populations	2,85953 Va	72,67%	0,7267*
Within populations	1,07566 Vb	27,33%	

^*^P < 0.05.

## Discussion

The epidemiology of *Hepatozoon* sp. in wild and domestic animals is still not fully elucidated around the world. The role of rodents in the epidemiology of *Hepatozoon* sp. should be further investigated to evaluate if genotypes circulating in rodents are specific of this group of mammals or might also circulate in wild carnivores and reptiles; in the latter case, rodents would play a role as intermediate or paratenic hosts in the epidemiological cycles.

The present study showed the occurrence of *Hepatozoon* spp. in several rodent species trapped in five Brazilian biomes: Cerrado, Pantanal, Amazon, Caatinga, and Atlantic Forest. *Hepatozoon* sp. DNA was detected in 24 different rodent genera in one or both PCR protocols based on the 18S rRNA gene. The percentage of positive animals and genera found in the present study was superior to that reported in previous works performed in Brazil^9,10^. This study provides the first report of the occurrence of *Hepatozoon* spp. in *Rattus rattus*, *Mus musculus*, *Proechimys roberti*, *P*. *cuvieri*, *Galea spixii*, *Hylaeamys megacephalus*, *Gracilinanus agilis*, *Cerradomys scotti*, *C*. *akroai*, *C*. *marinhus*, and *Wiedomys cerradensis*. Also, the positivity of rodents for *Hepatozoon* spp. was reported, for the first time, in the states of Bahia and Ceará, northeastern Brazil, Santa Catarina (South), Rio de Janeiro (Southeast), and Tocantins (central-western). Herein, a moderately high positivity for *Hepatozoon* was found among trapped rodents, with a description of the parasitism, for the first time, in certain species and localities not previously investigated. It is likely that the wide sampling, with 472 rodents collected in five Brazilian biomes, with diverse climatic and environmental characteristics, has influenced these results.

The sequences detected in rodents in the present study presented high *query coverage* and identity values (98–100%) for sequences of *Hepatozoon* spp. detected in rodents, reptiles, and ticks previously described in Brazil and other countries. BLAST and phylogeny (inferred by ML, Bayesian and Splitstree) analyses yielded similar results for both 18S rRNA protocols (analyzed separately and concatenated). The sequences detected in rodents in the present study were positioned in a large clade comprising *Hepatozoon* sequences previously detected in rodents and reptiles. *Hepatozoon* sequences from felids and canids were grouped in another large clade. These results corroborate with previous studies. *Hepatozoon* sequences detected in rodents in Slovakia and the Czech Republic were phylogenetically related to sequences detected in lizards and snakes, positioning apart from *H*. *canis* detected in dogs^17^. Similar results were obtained by Sousa^10^ when analyzing *Hepatozoon* sequences from rodents sampled in Brazilian Pantanal. These results suggest that rodents might play an important role as intermediate or paratenic hosts for *Hepatozoon* infections in reptiles; in contrast, these mammals do not seem to participate in the epidemiological cycles of *Hepatozoon* species parasitizing domestic and wild canids and felids in Brazil. In South Africa, one sequence of 18S rRNA *Hepatozoon* sp. detected in *Vulpes pallida* was closely related to *Hepatozoon* sequences detected in rodents (*Jaculus* sp.), reptiles, and marsupials from other localities^7^. Therefore, future studies should be conducted to investigate the real role of rodents in the epidemiology of canine hepatozoonosis in the African continent.

Haplotype diversity is controlled by multiple processes, such as mutation, recombination, and demography^18^. The haplotype diversity found in rodent-associated *Hepatozoon* 18S rRNA sequences in the present study was higher than those found in previous studies performed in Brazil. For instance, Gomes^19^ found four *Hepatozoon* 18S rRNA haplotypes in capybaras (*Hydrochoerus hydrochaeris*) in Marajó Island, northern Brazil. In Pantanal wetland, central-western Brazil, three *Hepatozoon* 18S rRNA haplotypes were found in rodents (*T*. *fosteri*) based on Ujvari’s PCR protocol^14^. When the same analysis was performed using *Hepatozoon* 18S rDNA sequences originated from Perkins and Keller’s PCR protocol^13^, four haplotypes were detected^10^. In the present study, among sequences obtained from Perkins and Keller’s PCR protocol^13^, only two haplotypes were detected, and among 17 sequences obtained from Ujvari’s PCR protocol^14^, five haplotypes were detected. Based on these results, we suggest that the *Hepatozoon* 18S rDNA haplotype analyzed in rodents should be performed based on sequences obtained from Ujvari’s PCR protocol^14^.

Unlike in previous studies, the present work analyzed the diversity of 18S rDNA haplotypes of *Hepatozoon* spp. described up to now in Brazil. For this purpose, 26 sequences from different localities were chosen from the present study (Rio de Janeiro, São Paulo, Ceará, Bahia, Mato Grosso State) and previous studies performed in the states of São Paulo^9,11^, Mato Grosso^8^, and Mato Grosso do Sul^10^. As a result, six haplotypes were detected among the 26 sequences, showing a significant heterogeneity of *Hepatozoon* sp. parasitizing this group of mammals. The existence of two large haplogroups between the 26 sequences was noticed as part of the TCS analysis. The two haplogroups presented a high level of genetic structure when compared in AMOVA. The FST value (0.7267), which was statistically significant, was sufficient to conclude that there is a difference between these two haplogroups; FST values higher than 0.25 characterize a strong pattern of genetic structuring^20^. Additionally, sequences detected in rodents from Mato Grosso do Sul were grouped separately, forming another haplogroup. Apparently, there are two distinct groups of haplotypes circulating in rodents in Brazil, one from the coastal region and Mato Grosso state and another from the central region of Brazil, with the sequences from Mato Grosso do Sul being relatively more distinct. To expand this primary analysis, new studies in Brazil should be performed, covering a larger number of rodent species and geographic region.

Until recently, 18S rDNA gene sequences comprised the only molecular markers for the analysis of *Hepatozoon* species diversity^21^. Although the 18S rDNA gene has been commonly used as a molecular marker for phylogenetic analyses, problems in separating closely related species might occur due to its high degree of conservation^22^. Mitochondrial genomes have been used to help unravel the complex phylogenetic relationship of piroplasmids^23^. Similarly, *H*. *canis* mitochondrial genome sequences have been recently described using next-generation sequence approaches^24^. Therefore, mitochondrial genomes should be used in the future as a barcode for discriminating *Hepatozoon* haplotypes and species that are shown to be closely related in phylogenetic inferences based on 18S rRNA. Additionally, the complete genome of *H*. *canis*^24^ will open opportunities to identifying novel target genes, allowing better discrimination of *Hepatozoon* haplotypes circulating in different animal species and vectors. Such an approach would contribute to a better definition/description of *Hepatozoon* species, epidemiological cycles, and trophic relationships.

## Methods

Sampling procedures were approved by the “Brazilian Institute for Environment and Natural Renewable Resources” (IBAMA) (IBAMA/CGFAU/LIC 3665-1) and by the Ethics Committee of Oswaldo Cruz Foundation (FIOCRUZ) (CEUA: P0007-99; P0179-03; P0292/06; L0015-07) and all experiments were performed in accordance with CGFAU and CEUA FIOCRUZ guidelines.

**Figure 6:**
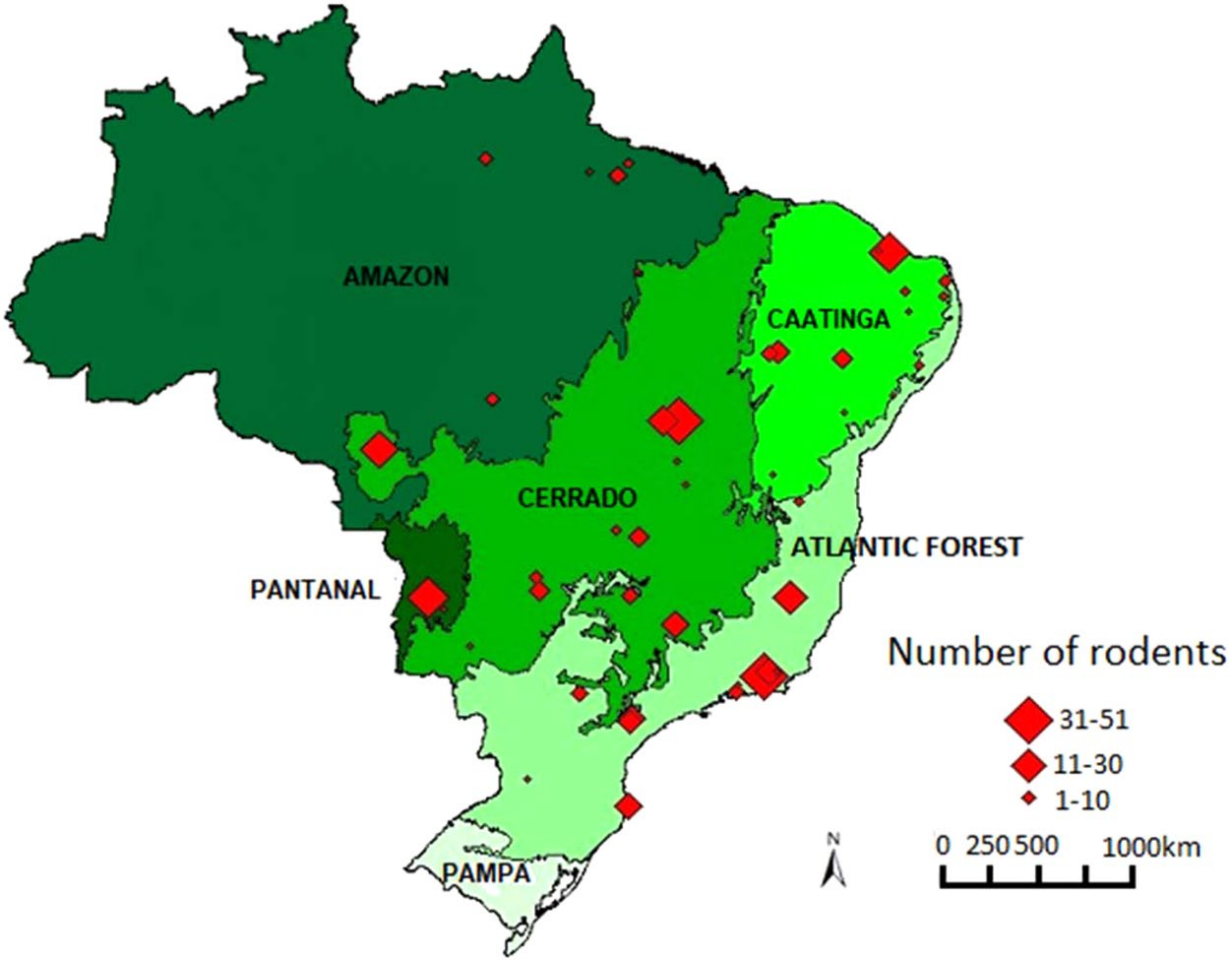
Distribution of trapped rodents (n = 472) in five Brazilian biomes.

Between 2000 and 2011, multiple rodent genera [n = 31] were trapped in five Brazilian biomes: Amazon, Cerrado, Atlantic Forest, Caatinga, and Pantanal (Fig. 6)^25^. Sampling places were chosen by convenience. Animals were caught using Tomahawk and Sherman “live-traps” during previous studies performed by the Laboratories of Trypanosomatid Biology and Biology and Parasitology of Wild Mammals Reservoirs Laboratories, Oswaldo Cruz Institute, Rio de Janeiro, Brazil^26,27^. Euthanasia of the sampled mammals was performed for taxonomic identification and/or diagnosis of parasites. Rodents were exsanguinated after anesthesia with intramuscular ketamine hydrochloride (100 mg/mL) and acepromazine (10 mg/mL), and euthanasia was assured by intracardiac injection of potassium chloride (19.1%, 2 mL/kg).

Spleen tissues from 472 rodents were collected and stored in DNase- and RNase-free microtubes containing ethanol and maintained at −20 °C until DNA extraction. DNA was extracted from 10 mg of each rodent spleen tissue using the DNeasy Blood and Tissue Kit (Qiagen^®^, Valencia, California, USA), according to manufacturer’s instructions. The DNA concentration and absorbance ratio (260/280 nm) were measured using a spectrophotometer (Nanodrop, Thermo Scientific, USA).

To evaluate the quality of the extracted DNA, each spleen DNA sample was tested by a conventional cPCR targeting the mammal *irbp* (“interphotoreceptor retinoid-binding protein”) gene as an internal control^28^. Samples negative for the above PCR protocol were tested in another cPCR targeting the mammal *gapdh* gene^29^. Samples negative for both protocols were removed from subsequent analyses.

Two different PCR protocols were used aiming at amplifying different regions of 18SrRNA of *Hepatozoon* spp. based on the Perkins and Keller (targeting a fragment of 800 bp)^13^ and Ujvari^14^ (targeting a fragment of 600 bp) protocols. Rodents were considered positive for *Hepatozoon* spp. when samples amplified the target region in one or both protocols. In samples positive for both of the above PCR protocols, the two *Hepatozoon* 18S rRNA sequences obtained were concatenated to obtain a large 18S rRNA fragment to be used in phylogenetic analyses (approximately 1400 bp). *Hepatozoon caimani* DNA obtained from a naturally infected *Caiman crocodilus yacare*^30^ was used as a positive control. Ultra-pure sterile water (Life Technologies^®^, Carlsbad, CA, USA) was used as a negative control in all PCR assays. The results were visualized in 1% agarose gel stained by ethidium bromide solution. Only amplicons showing high-intensity bands in agarose gel electrophoresis were sequenced. The amplified products were purified using the Silica Bead DNA gel extraction kit (Thermo Fisher Scientific^®^, Waltham, MA, USA), following the manufacturer’s protocol. The sequencing of the two different regions of 18S rRNA *Hepatozoon* gene fragments was carried out using ABI PRISM 310DNA Analyzer (Applied Biosystems^®^, Foster City, CA, EUA)^31^. The quality of the obtained sequence electropherograms was checked by Phred-Phrap software version 23, and the quality of each nucleotide sequence was observed^32,33^. Each nucleotide was checked for a score and was considered of good quality when scoring Phred >20. Additionally, the presence of a double read in each nucleotide was evaluated. Consensus sequences obtained by the alignment of the sense and antisense sequences were constructed using the same software^34^. The BLAST program was used to analyze the sequences of nucleotides, aiming to browse and compare with sequences from the GenBank international database^35^. All sequences that showed appropriate quality standards (query coverage >90%) and identity with *Hepatozoon* spp. were deposited in GenBank. Samples showing positive results for both PCR protocols had their sequences concatenated. The obtained sequences were aligned with those retrieved from GenBank using MAFFT software, version 7^15^. Sequences used for phylogenetic inferences were selected from BLAST results and other studies performed in Brazil and other countries (Supplementary Information, Table 2). The Bayesian inference (BI) analysis was performed with MrBayes 3.1.2^36^. Markov chain Monte Carlo (MCMC) simulations were run for 10^6^ generations with a sampling frequency of every 100 generations and a burn-in of 25%. The number of generations was selected based on the value of the average standard deviation of split frequencies (between 0.01 and 0.05 according to MrBayes version 3.2 Manual) (Ronquist, Huelsenbeck, Teslenko 2011). The best model of evolution was selected by the program jModelTest2 (version 2.1.6) on 11 XSEDE^19^, under the Akaike Information Criterion (AIC)^37^. ML analysis was performed with cluster Blackbox RaxML^38^. All phylogenetic analyses were performed using the CIPRES Science Gateway^39^. The phylogenetic tree edition and rooting (outgroup) were performed using the Treegraph 2.0 beta software^40^. Nucleotide sequence genealogies were inferred by *Network* analyzes using Splitstree v4.11.3 software using sequences detected in the present study and sequences deposited in GenBank from other studies^41^. A haplotypic network using the software TCS^16^ with a 95% parsimony threshold was performed to infer the distance between haplotypes and connection between its occurrence and geographic areas. The molecular variance (AMOVA) analysis was performed using Arlequin v.3.11^42^, including all haplogroups observed in the haplotypic network.

### Ethics statement

Sampling procedures were approved by the “Brazilian Institute for Environment and Natural Renewable Resources” (IBAMA) (IBAMA/CGFAU/LIC 3665-1) and by the Ethics Committee of Oswaldo Cruz Foundation (FIOCRUZ) (P0007-99; P0179-03; P0292/06; L0015-07).

## Supplementary information

Supplementary info
