# Supplementary material for: Genetic diversity of Hepatozoon spp. in rodents from Brazil
Source: Sci Rep. 2019 Jul 12;9:10122. doi: 10.1038/s41598-019-46662-2 (PMC6626033; doi:10.1038/s41598-019-46662-2)

**Genetic diversity of *Hepatozoon* spp. in rodents from Brazil**

Perles, L.^1^; Roque, A.L.R^2^; D’Andrea, P.S.^3^; Lemos, E.R.S.^4^; Santos, A.F.^5^; Morales, A.C.^5^; Machado, R.Z.^1^; André, M.R^1*^

^1^Laboratório de Imunoparasitologia, Departamento de Patologia Veterinária, Universidade Estadual “Júlio de Mesquita Filho”, Jaboticabal, Brasil.  
^2^Laboratório de Biologia de Tripanosomatídeos, Instituto Oswaldo Cruz/Fiocruz, Rio de Janeiro, Brasil.
^3^Laboratório de Biologia e Parasitologia de Mamíferos Silvestres Reservatórios, Instituto Oswaldo Cruz/Fiocruz, Rio de Janeiro, Brasil.
^4^Laboratório de Hantaviroses e Rickettsioses, Instituto Oswaldo Cruz/Fiocruz, Rio de Janeiro,Brasil.
^5^Departamento de Biologia Aplicada à Agropecuária, Universidade Estadual “Júlio de Mesquita Filho”, Jaboticabal, Brasil.  

**Table 1.** Total number and rodents species positive for *Hepatozoon* sp. using the protocols of Perkins and Keller (2001) and Ujvari et al., (2004) based on the 18SrRNA gene, according to rodent species and respective states and biomes of origin.

| **Biome/States** | | **Total of rodents sampled in each biome** | **Species samples (n)** | **Total of positive samples**  **Perkins and Keller (2001)** | **Positive rodent species**  **Perkins e Keller (2001)** | **Total of positive samples**  **Ujvari et al. (2004)** | **Positive rodent species**  **Uvjari et al. (2004)** |
| --- | --- | --- | --- | --- | --- | --- | --- |
| **Caatinga**  Piauí, Ceará, Bahia, Rio Grande do Norte | 80 | *Galea spixxi*: 19  *Mus musculus*: 5  *Necromys lasiurus*: 1  *Rattus rattus*: 12  *Rhipidomys macrurus*: 7  *Thrichomys laurentius*: 28  *Thrichomys inermis*: 7  *Wiedomys cerradensis*: 1 | 11 | *Galea spixxi*: 4  *Rattus rattus*: 1  *Thrichomys laurentius:* 2  *Thrichomys inermis*: 3  *Wiedomys cerradensis*: 1 | 28 | *Galea spixxi:* 6  *Mus musculus:* 2  *Necromys lasiurus:* 1  *Rattus rattus:* 4  *Rhipidomys macrurus:* 2  *Thrichomys laurentius:* 9  *Thrichomys inermis:* 3  *Wiedomys cerradensis:* 1 |  |
| **Atlantic Forest**  São Paulo, Rio de Janeiro, Santa Catarina | 122 | *Akodon* sp.: 6  *Akodon montesis*: 35  *Akodon cursor*: 11  *Brucepattersonius iheringi*: 1  *Calomys tener*: 8  *Coendou* sp.: 2  *Delomys dorsalis*: 8  *Euryoryzomys russatus*: 11  *Guerlinguetus brasiliensis*: 1  *Mus musculus*: 3  *Necromys lasiurus*: 2  *Nectomys squamipes*: 3  *Oligoryzomys nigripes*: 16  *Oligoryzomys flavescens*: 3  *Oxymycterus* sp.: 4  *Oxymycterus dasythricus*: 2  *Trinomys dimidiatus:* 4  *Trinomys gratiosus bonafidei:* 1  *Sooretamys angouya*:1 | 36 | *Akodon cursor*: 6  *Akodon montensis*: 19  *Delomys dorsalis*: 2  *Euryoryzomys russatus*: 2  *Mus musculus*: 1  *Necromys lasiurus*: 2  *Oligoryzomys nigripes*: 4 | 51 | *Akodon cursor*: 10  *Akodon montensis*: 17  *Akodon* sp.: 1  *Calomys tener*: 2  *Delomys dorsalis*: 2  *Euryoryzomys russatus*: 3  *Mus musculus*: 2  *Oligoryzomys nigripes*: 8  *Nectomys squamipes*: 2  *Trinomys dimidiatus*: 3  *Trinomys gratiosus bonafidei*: 1 |  |
| **Pantanal**  Mato Grosso do Sul | 53 | *Holochilus chacarius:* 3  *Calomys expulsus*: 1  *Thrichomys fosteri*: 16  *Necromys lasiurus:* 6  *Clyomys laticeps*: 20  *Oecomys marmorae*: 2  *Nectomys rattus*: 2  *Nectomys squamipes*: 3 | 2 | *Calomys expulsus:* 1  *Thrichomys fosteri*: 1 | 18 | *Calomys expulsus:* 1  *Thrichomys fosteri:* 7  *Necromys lasiurus:* 2  *Clyomys laticeps:* 4  *Nectomys rattus:* 2  *Nectomys squamipes:* 2 |  |
| **Cerrado**  Minas Gerais, Goiás | 89 | *Akodon lindberghi*: 2  *Akodon montensis*: 4  *Calomys cerqueirae*: 3  *Calomys expulsus*: 1  *Calomys tener*: 1  *Cavia* sp.: 1  *Cerradomys maracajuensis*: 1  *Cerradomys marinhus*: 2  *Cerradomys scotti*: 1  *Cerradomys* sp.: 1  *Cerradomys subflavus*: 1  *Gracilinanus agilis*: 2  *Hylaeamys megacephalus:* 4  *Necromys lasiurus*: 17  *Nectomys rattus*: 6  *Nectomys squamipes*: 5  *Oecomys catherinae*: 1  *Oecomys* *bicolor:* 2  *Oecomys* sp.: 2  *Oligoryzomys nigripes*: 10  *Oxymycterus delator*: 2  *Rhipidomys macrurus:* 9  *Thrichomys apereoides*: 10 | 3 | *Oecomys bicolor:* 1  *Oligoryzomys nigripes:* 2 | 19 | *Cerradomys marinhus*: 1  *Necromys lasiurus*: 4  *Necromys squamipes*: 2  *Nectomys rattus*: 1  *Oligoryzomys nigripes*: 3  *Rhipidomys macrurus*: 4  *Thrichomys apereoides*: 4 |  |
| **Amazon**  Tocantins, Pará, Mato Grosso | 128 | *Zygodontomys* sp.: 3  *Thrichomys inermis*: 1  *Rhipidomys* sp.: 1  *Rhipidomys macrurus*: 1  *Rattus rattus*: 10  *Proechimys* sp.: 1  *Proechimys roberti*: 5  *Proechimys cuvieri*: 8  *Proechimys goeldii*: 3  *Oligoryzomys* sp.: 3  *Oligoryzomys moojeni*: 3  *Oligoryzomys mattogrossae*: 3  *Oecomys catherinae*: 2  *Necromys lenguarum*: 14  *Necromys lasiurus:* 11  *Mus musculus*: 2  *Hylaeamys megacephalus*: 16  *Cerradomys akroai*: 4  *Calomys* sp. : 8  *Calomys expulsus*: 17  *Calomys callidus*: 12 | 17 | *Zygodontomys sp.:* 1  *Rhipidomys sp.:* 1  *Rattus rattus:* 1  *Proechimys roberti:* 1*Proechimys cuvieri:* 1*Oligoryzomys sp.:* 1*Oligoryzomys moojeni:* 1*Necromys lenguarum:* 3*Hylaeamys megacephalus:* 1  *Calomys expulsus*: 2*Calomys callidus*: 4 | 56 | *Zygodontomys* sp.: 1  *Thrichomys inermis*: 1  *Rhipidomys* sp.: 1  *Rattus rattus*: 3  *Proechimys* sp.: 1  *Proechimys roberti*: 2  *Proechimys cuvieri*: 2  *Oligoryzomys* sp.: 3  *Oligoryzomys moojeni*: 3  *Oligoryzomys mattogrossae*: 2  *Oecomys catherinae*: 1  *Necromys lenguarum*: 5  *Necromys lasiurus*: 2  *Mus musculus*: 2  *Hylaeamys megacephalus*: 8  *Cerradomys akroai*: 4  *Calomys* sp.: 3  *Calomys expulsus*: 10  *Calomys callidus*: 2 |  |

**Table 2.** Sequences used for phylogenetic inferences selected from Blast results and from other studies performed in Brazil and in other countries with GenBank accession number, *Hepatozoon* species, host, country and reference.

| **GenBank accession number** | ***Hepatozoon* species** | **Host** | **Country** | **Reference** |
| --- | --- | --- | --- | --- |
| KU667308 | *Hepatozoon milleri* | *Akodon montensis* | Brazil | Demoner et al., 2018 |
| FJ719815 | *Hepatozoon* sp. | *Abrothrix olivaceus* | Chile | Merino et al., 2009 |
| KX776354 | *Hepatozoon* sp. | *Thylamys macrurus* | Brazil | Sousa et al., 2017 |
| KX776353 | *Hepatozoon* sp. | *Oecomys marmorae* | Brazil | Sousa et al., 2017 |
| KX453646 | *Hepatozoon* sp. | *Psammophis schokari* | Oman | Maia et al., 2016 |
| JX644996 | *Hepatozoon* sp. | *Myodes glareolus* | Hungary | Rigo et al., 2002 |
| KU597253 | *Hepatozoon* sp. | *Myodes glareolus* | Slovakia | Hamsikova et al., 2016 |
| KM234617 | *Hepatozoon* sp. | *Hemidactylus mabouia* | Brazil | Harris et al., 2004 |
| KF939627 | *Hepatozoon* sp. | *Elaphe carinata* | China | Wu et al., 2014 |
| AY252103 | *Hepatozoon* sp. | *Liasis fuscus* | Australia | Uvjari et al., 2005 |
| MF322539 | *Hepatozoon caimani* | *Caiman crocodilus yacare* | Brazil | Bouer et al., 2017 |
| KM234615 | *Hepatozoon* sp. | *Hemidactylus mabouia* | Brazil | Harris et al., 2004 |
| JF491242 | *Hepatozoon* sp. | *Boa constrictor* | USA | Allen et al., 2011 |
| KX387860 | *Hepatozoon* sp. | *Phymaturus calcogaster* | Spain | Garcia-Roa et al., 2017 |
| KC342526 | *Hepatozoon* sp. | *Crotalus durissus terrificus* | Brazil | O'Dwyer et al., 2013 |
| KF989489 | *Hepatozoon* sp. | *Vulpes vulpes* | USA | Kisler et al., 2014 |
| KJ499515 | *Hepatozoon* sp. | *Vulpes rueppellii* | África | Maia et al., 2014 |
| KP167594 | *Hepatozoon canis* | *Amblyomma sculptum* | Brazil | Melo et al., 2015 |
| KJ831221 | *Hepatozoon canis* | *Canis lupus familiaris* | Brazil | Melo et al., 2015 |
| GQ176285 | *Hepatozoon canis* | *Canis lupus familiaris* | Brazil | Pereira et al., 2009 |
| FJ943578 | *Hepatozoon canis* | *Canis lupus familiaris* | Brazil | Pereira et al., 2009 |
| MG241229 | *Hepatozoon canis* | *Rhipicephalus sanguineus* | Brazil | Santos et al., 2017 |
| KX816958 | *Hepatozoon canis* | *Lycalopex gymnocercus* | Brazil | Silva et al., 2017 |
| AY461376 | *Hepatozoon canis* | *Pseudalopex gymnocercus* | Brazil | Criado-Fornelio et al., 2006 |
| KU955997 | *Hepatozoon sp.* | *Vulpes chama* | South Africa | Harris et al., 2016 |
| JF491228 | *Hepatozoon sp.* | *Felis catus* | USA | Allen et al., 2011 |
| EU249992 | *Hepatozoon americanum* | *Sigmodon hispidus* | USA | Allen et al., 2008 |
| JX415175 | *Hepatozoon americanum* | *Canis latrans* | USA | Starkey et al., 2012 |
| KU729739 | *Hepatozoon americanum* | *Canis lupus familiaris* | Brazil | Gomes et al., 2016 |
| KX757032 | *Hepatozoon silvestris* | *Felis silvestris silvestris* | Bosnia | Hodzik et al., 2016 |
| KU232308 | *Hepatozoon felis* | *Panthera onca* | Brazil | Furtado et al., 2016 |
| KP410283 | *Hepatozoon sp.* | *Felis catus* | Brazil | André et al., 2015 |
| KJ499537 | *Hepatozoon sp.* | *Jaculus jaculus* | África | Maia et al., 2015 |
| KC342527 | *Hepatozoon sp.* | *Crotalus durissus terrificus* | Brazil | O'Dwyer et al., 2013 |
| KC342526 | *Hepatozoon sp.* | *Crotalus durissus terrificus* | Brazil | O'Dwyer et al., 2013 |
| KX387861 | *Hepatozoon sp.* | *Phymaturus calcogaster* | Spain | Garcia-Roa et al., 2017 |
| FJ876447 | *Hepatozoon sp.* | *Speothus venaticus* | Brazil | André et al., 2009 |

**Figure 1**. Phylogenetic tree based on an alignment of 1400 bp fragment (concatenated analysis) of *Hepatozoon* spp. 18SrRNA sequences, using Maximum likelihood inference (ML) method and TIM+G evolutionary model. Numbers at nodes correspond to bootstrap. Accession numbers are indicated in the sequences. Sequences of *Hepatozoon* spp. detected in the present study are highlighted in red.


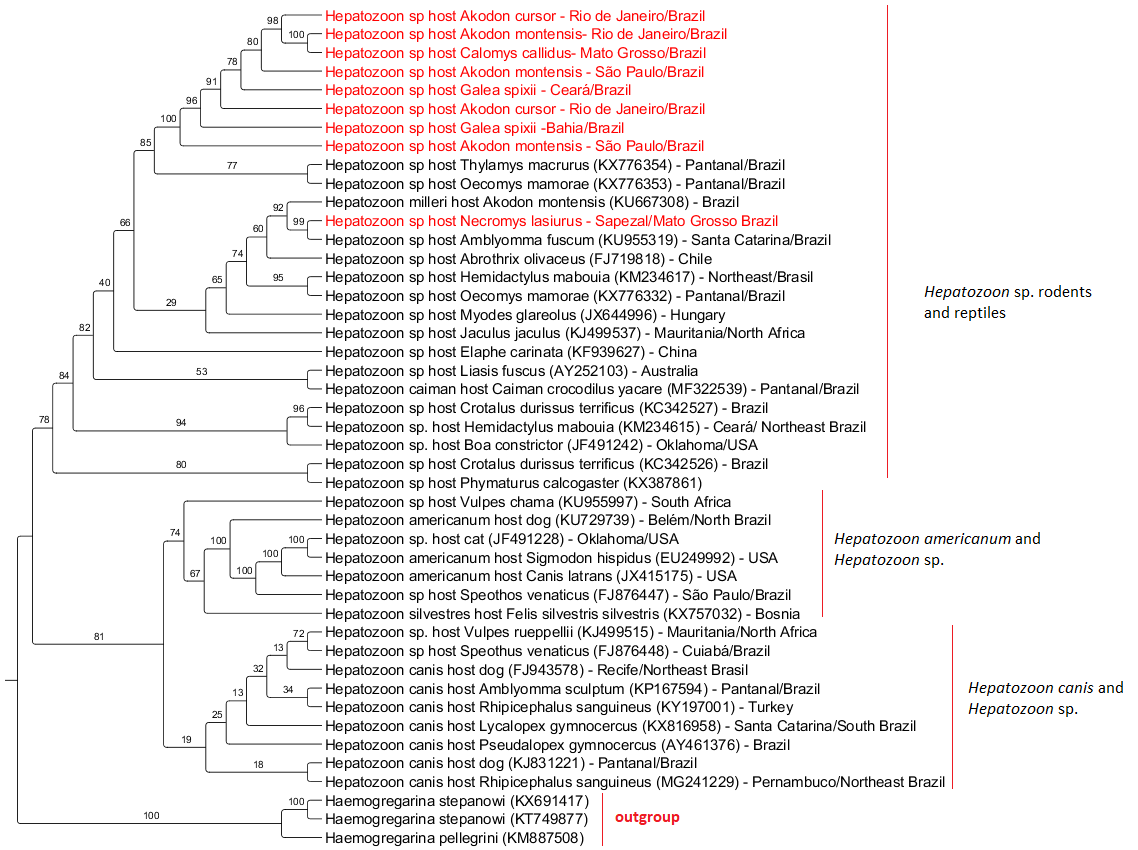

Supplement: Supplementary file 1 — Supplementary info [file 41598_2019_46662_MOESM1_ESM.docx]
